# Influence of MoO_3_(110) Crystalline Plane on Its Self-Charging Photoelectrochemical Properties

**DOI:** 10.1038/srep07428

**Published:** 2014-12-11

**Authors:** Shi Nee Lou, Nicholas Yap, Jason Scott, Rose Amal, Yun Hau Ng

**Affiliations:** 1 Particles and Catalysis Research Group, School of Chemical Engineering, The University of New South Wales, Sydney NSW 2052, Australia

## Abstract

Nanocrystalline molybdenum oxide (α-MoO_3_) thin films with iso-oriented crystalline layers were synthesised by the anodisation of Mo foils. Upon band-gap excitation using light illumination, α-MoO_3_ generates excited electrons for reductive reactions and stores some of the excited electrons in its layered crystalline structure via alkali cation intercalation. These stored electrons can be subsequently discharged from α-MoO_3_ to allow reductive reactions to continue to occur under non-illuminated conditions. The modulation of water concentrations in the organic/aqueous anodisation electrolytes readily produces α-MoO_3_ crystals with high degree of (kk0) crystallographic orientation. Moreover, these (kk0)-oriented MoO_3_ crystals exhibit well-developed {hk0} and {0k0} crystal facets. In this paper, we show the benefits of producing α-MoO_3_ thin films with defined crystal facets and an iso-oriented layered structure for in situ storing of excited charges. α-MoO_3_ crystals with dominant (kk0) planes can achieve fast charging and a strong balance between charge release for immediate exploitation under illuminated conditions and charge storage for subsequent utilisation in dark. In comparison, α-MoO_3_ crystals with dominant (0k0) planes show a preference for excited charge storage.

Photocatalysis is important for converting solar energy into more utilisable forms of energy such as H_2_ fuel and electricity^1^,^2^,^3^. To date, TiO_2_ is the benchmark semiconductor photocatalyst due to its chemical stability and high activity. Recently, photocatalysts such as Fe_2_O_3_, BiVO_4_, ZnO and CdS have also received significant research attention due to their distinct properties (e.g. high carrier mobilities, suitable bandgap, chemical stability and long carrier lifetimes)^4^,^5^,^6^,^7^,^8^. However, a major limitation of these traditional photocatalysts is that they function under light only. If the source of light is withdrawn, excited electrons and holes will recombine instantly and disrupt any on-going photocatalytic activities. Research strategies to extend the applicability of solar energy utilisation to non-illuminated (i.e. dark) conditions are currently realised by dual but separate material systems for light harvesting and energy storage, respectively. For instance, photocatalysts with reductive energy storage abilities have been developed by coupling photocatalysts e.g. TiO_2_, with energy storage materials e.g. WO_3_ or MoO_3_, so that surplus electrons from UV-irradiated TiO_2_ can be transferred and stored in the conduction band of WO_3_ or MoO_3_^9^,^10^. The stored electrons are subsequently discharged from WO_3_ or MoO_3_ in the dark for specific reaction(s). A second example involves a dye-sensitized solar cell being modified into a photovoltaically self-charging battery by incorporating a charge-storage layer (e.g. WO_3_) underneath the photoactive layer (dye-coated TiO_2_)^11^. Upon illumination, excited electrons generated in the dye are injected into TiO_2_ and then WO_3_ for electron storage. Under the discharging condition, the stored electrons are discharged from WO_3_ via an external load to the Pt electrode. In this manner, the device acts as a solar cell during illumination and a battery after illumination.

In general, the drawbacks of existing light harvesting and energy storage systems are: (i) the need for an external electron source for electron storage (i.e. TiO_2_ or dye); (ii) the requirement of multi-component, hybrid photoelectrode composites; and (iii) the limited efficiency arising from the possible electron losses during the charge transfer process. To overcome the above limitations, our group has recently reported the self-photo-recharge ability of WO_3_^12^,^13^ and MoO_3_^14^ photocatalysts as illustrated in Figure 1 (for MoO_3_). Both WO_3_ and MoO_3_ photocatalysts are distinguished from traditional photocatalysts by their ability to also act as an advanced energy storage material. Owing to an open crystalline structure, WO_3_ and MoO_3_ are widely exploited as intercalation host materials for high energy density Li-ion batteries. In addition, unlike most intercalation transition metal oxide compounds which are not photosensitive, both WO_3_ and MoO_3_ possess an optical band gap and thus can directly harvest solar energy. The union of an intercalation host structure and an optical band structure enable both WO_3_ and MoO_3_ to generate excited charges for reductive reactions upon photo-excitation and store a portion of the excited charges in their crystal structures via tungsten or molybdenum bronze formation. The stored charges can be subsequently released in an on-demand manner for utilisation in the dark, akin to a self-photo-rechargeable photoelectrochemical (PEC) cell. Therefore, WO_3_ and MoO_3_ as photocatalysts exhibit the potential to combine solar energy conversion and charge storage in a single-material system.

As depicted in Figure 1, the crystal structure of orthorhombic-phase MoO_3_ (α-MoO_3_) comprises heavily distorted MoO_6_ octahedra connected via edges and corners in zig-zag chains to form a double layer planar structure. Successive double layers are stacked and held together by weak van der Waals forces along [010]. Ion intercalation into the interlayer gaps of the structure is strongly facilitated by the weakly interacting double layers. We recently established the dependence of the excited charge storage and release under light and dark conditions, respectively, was governed by the crystallography of MoO_3_^14^. A more distorted MoO_6_ unit cell was found to be effective for photoexcited charge generation as the dipole moment caused by the varied bond lengths in the octahedra aided electron-hole pair separation. In addition, MoO_3_ thin films with highly-oriented (0k0) planes exhibited enhanced excited charge storage capacities compared with both non-(0k0)-oriented-crystalline MoO_3_ and less crystalline-(0k0)-oriented MoO_3_. The enhanced charge storage capacities of crystalline MoO_3_ thin films with highly-oriented (0k0) planes were attributed to the presence of numerous interlayer lattice planes and the long-range order of the structure for rich alkali cation intercalation.

The crystallographic orientation of the MoO_3_ crystals was found to exert a strong influence on the excited charge storage capacity of the MoO_3_-based PEC cell. The (0k0)-oriented MoO_3_ crystals in previous work showed a strong preference for excited charge storage. In view of the intermittent nature of the sun's energy, this implies an MoO_3_-based PEC cell intensely-oriented in the (0k0) planes is particularly effective for harvesting and storing solar energy in daylight hours and the stored solar energy can be subsequently released for utilisation in the dark hours on demand. To further improve the utilisation efficiency of the solar energy, an MoO_3_-based PEC cell that can achieve a strong balance between excited charge release for immediate exploitation under illuminated conditions and charge storage for subsequent utilisation in the dark is very captivating.

In this work, we further investigate the influence of MoO_3_ crystallography on excited charge storage and release using crystalline MoO_3_ thin films with iso-oriented (kk0) crystalline planes. Through fine-controlling of the MoO_3_ crystal structure orientation, our goal is to develop a nanostructured MoO_3_ thin film that regulates and balances the level of charge release for immediate usage and charge storage for later utilisation in the dark. The MoO_3_ films with dominant (kk0) planes were fabricated via the anodisation of Mo foils in fluoride-containing ethylene glycol electrolytes with varied water concentrations. The resultant MoO_3_ thin films were found to possess an increasing number of (kk0) planes with higher water concentration. The material properties of the (kk0)-oriented MoO_3_ thin films and their charge storage performances under illuminated and non-illuminated conditions were probed. Compared to the (0k0)-oriented MoO_3_ crystals in our previous work, MoO_3_ thin films with dominant (kk0) crystals could achieve fast charging and a strong balance between anodic photocurrent generation and excited charge storage.

## Methods

Molybdenum (Mo) foil (Sigma-Aldrich, ≥99.99%, 0.1 mm thick) was anodised in 100 g of ethylene glycol solution comprising 0.5 wt% sodium fluoride (NaF, Sigma-Aldrich, 99.99%) and 1–10 wt% water. The molybdenum foils were degreased by sonicating in acetone, followed by rinsing with acetone, ethanol and distilled water. The degreased molybdenum foils were subsequently dried in an oven at 110°C. The pre-treated molybdenum foil was contacted with a Cu spring and pressed against an o-ring in the anodisation reactor, exposing 4.15 cm^2^ of molybdenum foil to the electrolyte solution. The anodisation reactor is a two-electrode cell comprising a platinum foil as the counter electrode (cathode) and the molybdenum foil as the working electrode (anode). The molybdenum foils were anodised at 40 V for 2 h in the as-prepared electrolytes with varied water concentrations. The as-synthesised MoO_3_ thin films were dried in a vacuum overnight and subsequently annealed in air at 450°C for 2 h with a ramping rate of 5°C per min. The samples were then cooled by natural air convection in the furnace back to the ambient condition.

### Characterisation

The crystalline structure of the α-MoO_3_ thin films was characterised by glancing angle X-ray diffraction using Cu Kα radiation (λ = 1.54 Å) with a potential of 40 kV and a current of 30 mA. Scanning electron micrographs (SEM) of the thin films were taken using a Hitachi S4500 system operating at an accelerating voltage of 10 kV. Raman spectra were recorded on a Reinshaw inVia spectrometer utilising a 514 nm wavelength Argon ion laser with 1800 lines/mm grating to assess chemical bonding in the α-MoO_3_. Diffuse reflection ultraviolet and visible (DRUV-vis) spectra of the samples were recorded using a Shimadzu UV-3600 Spectrophotometer in the range of 200–600 nm at a scan rate of 100 nm min^−1^. The reflectance of the sample was measured and the corresponding absorbance (F(R)) was calculated using Kubelka-Munk function. Photoluminescence emission was measured using a Jobin Yvin Technology Fluoromax-4 spectrofluorometer at an excitation wavelength of 250 nm and recorded over the range 300 nm to 450 nm.

### Photoelectrochemical measurements

Photocurrent measurements were taken in 0.1 M sodium sulphate (Na_2_SO_4_, Fluka, ≥99%) electrolyte at room temperature using an Autolab potentiostat (Model PGSTAT302N) at 1.0 V in a two-electrode PEC cell with Pt as the counter and reference electrode and α-MoO_3_ thin film as the working electrode. The electrolyte solution was purged with nitrogen gas for 10 min prior to measurement and the purging was continued for the duration of photocurrent measurements to remove any dissolved oxygen in the cell. The exposed surface area of the α-MoO_3_ electrode was 2 cm^−2^ for all PEC measurements. The samples were photo-charged under UV-illumination for 1.5 h. UV-illumination was provided by a 300 W Xe lamp (Perkin Elmer, CERMAX, LC-300BUV). To remove any heating of the electrolyte solution during UV-illumination, a water jacket was placed between the Xe lamp and PEC cell. Similar two-electrode systems were utilised to measure the α-MoO_3_ thin film electrodes' charge storage abilities. Photocharging was performed under the same conditions as the photocurrent measurements. Following the photocharging process, the charged α-MoO_3_ thin film electrodes were discharged in the dark for the same duration. The total amount of stored charge is reflected by the total amount of discharge (coulomb) recorded during the discharge process under dark conditions.

## Results and Discussion

### Crystal Structure and Morphology

**Figure 2:**
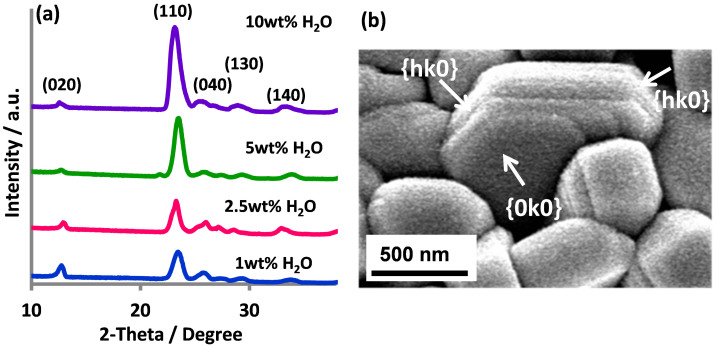
(a) XRD patterns of α-MoO_3_ thin films synthesised using 0.5 wt% NaF ethylene glycol electrolyte with varying water content and; (b) high magnification SEM images of α-MoO_3_ particles (5 wt% H_2_O).

Figure 2a shows the XRD patterns of the MoO_3_ thin films. The diffraction peaks are readily indexed to orthorhombic phase α-MoO_3_ (JCPDS card no. 05-0508). No peak representing other crystal phases or impurities was detected. The patterns consist of one strong (110) peak at 23° and several weak peaks corresponding to the (0k0) and other (hk0) planes, which differs from the typical pattern of α-MoO_3_^14^. Orthorhombic MoO_3_ is known to exhibit pronounced crystallographic anisotropy leading to growth rate differences in the three principle axes: [001] > [100] ≫ [010]^14^. As a result, α-MoO_3_ crystals tend to crystallise with a thin platelet morphology with well-developed (0k0) crystal faces while their long axis lays preferentially along the [001] direction^14^,^15^. XRD patterns of these α-MoO_3_ crystals generally reflect intense (0k0) diffraction peaks^14^,^15^. However, the α-MoO_3_ thin film samples obtained in this work exhibited a significantly enhanced I_(110)_/I_(020)_ XRD peak intensity ratio compared to the typical pattern of α-MoO_3_ which suggests the closed packed planes for these films are the (kk0) planes. The diminished (0k0) diffraction peaks further suggest the layered structure is less periodic in the [001] direction, which implies the layered structures of these (kk0)-oriented MoO_3_ films are constructed from smaller octahedral bi-layer sheets.

Figure 2b shows a high magnification SEM image of the particles obtained using the 5 wt% H_2_O electrolyte. The exhibited crystal facets of the α-MoO_3_ particles agree with the XRD observations. The α-MoO_3_ particles possess thick {0k0} plates with enlarged {hk0} side facets unlike typical α-MoO_3_ crystals^14^,^15^,^16^. For instance, in our earlier work, where Mo foil was anodised in an aqueous electrolyte containing 0.5 wt% NaF at 2.5 V for 20 min, the α-MoO_3_ particles were (0k0) crystal plane dominant and exhibited a flat MoO_3_{010} facet^14^. The change in crystal growth direction during anodisation appears to be invoked by the non-aqueous electrolyte environment^14^.

In general, the anodisation of Mo metal comprises two competing processes, the anodic oxidation process and the chemical dissolution process as shown by Eq. 1 and Eq. 2, respectively.


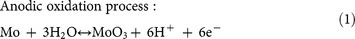

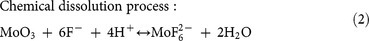


Upon the application of a constant voltage, the field-assisted oxidation of the Mo foil forms a compact oxide layer on the metal surface (Eq. 1). With prolonged anodisation, the anodisation current is observed to rise rapidly with time, indicating a strong rate of chemical dissolution at the metal oxide-electrolyte interface (Eq. 2). Compared to an aqueous electrolyte, anodic oxidation of the parent metal surface in a non-aqueous electrolyte tends to be relatively slow due to the lower oxygen content. As a result, chemical etching of the surface oxide layer by the fluoride etchant during anodisation is typically more pronounced in a non-aqueous electrolyte than in an aqueous electrolyte. The altered morphology of the α-MoO_3_ crystals with thick {0k0} plates and enlarged {hk0} side faces signifies fluoride etching of the α-MoO_3_ surface is highly preferential along [001] while crystallite growth has been reversed and forced along [010]^17^. The (kk0)-oriented crystalline layers of the α-MoO_3_ thin films are referred to the promoted growth along (kk0) planes within the crystal structure under our synthetic conditions. The presence of (kk0)-planes can be conveniently observed in almost every individual particle on the α-MoO_3_ thin film as shown in the SEM images (Figure S1) in the supporting information.

### Particle Size Distribution of MoO_3_ Thin Films with Varied Water Concentrations

**Figure 3:**
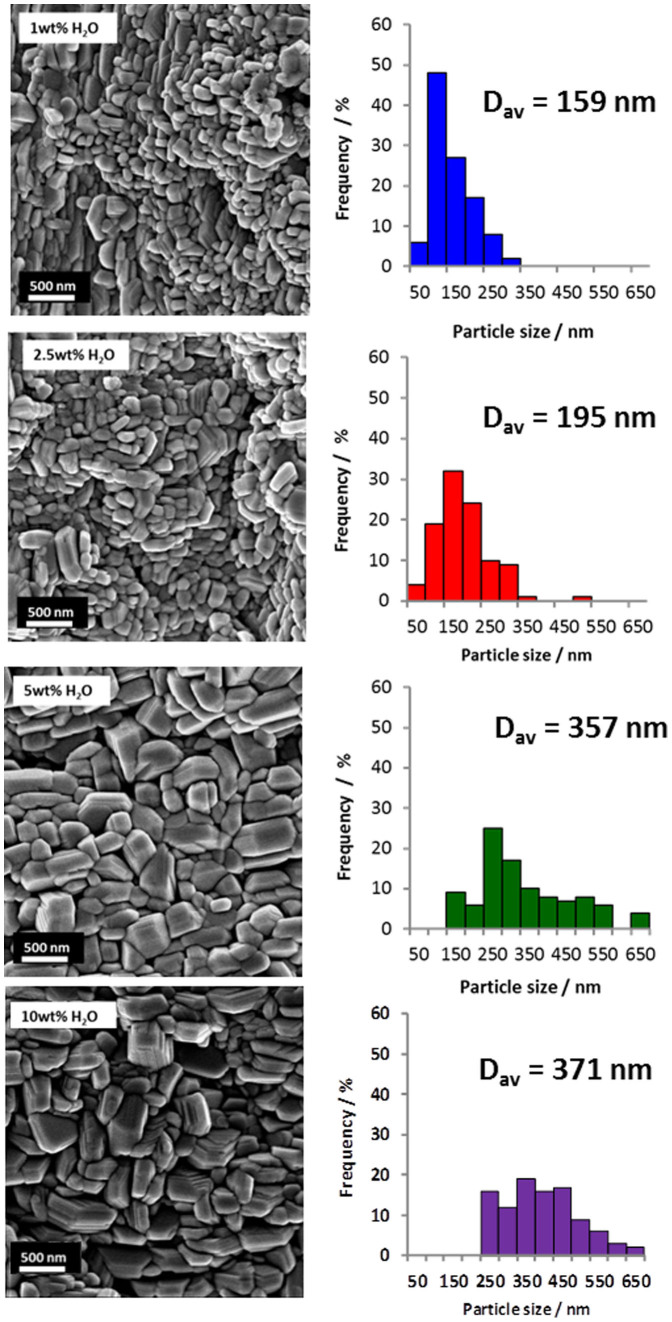
SEM images and particle size distributions of α-MoO_3_ thin films synthesised using varying water content.

Figure 3 shows the SEM images and estimated particle size distributions of the obtained α-MoO_3_ thin films with varying water concentrations. SEM analysis reveals a dense nanoparticle pattern on all film surfaces. The particle size was determined by averaging the longest length of 100 particles. The average particle sizes of the α-MoO_3_ thin films synthesised at different water concentrations are 159 nm (1 wt% H_2_O), 195 nm (2.5 wt% H_2_O), 357 nm (5 wt% H_2_O) and 371 nm (10 wt% H_2_O). The degree of chemical etching of the oxide is clearly manifested in the particle sizes where the higher fluoride coverage fraction, arising from the lower water concentration in the electrolyte, enhances the chemical etching rate leading to smaller crystalline particles.

### Optical Properties

**Figure 4:**
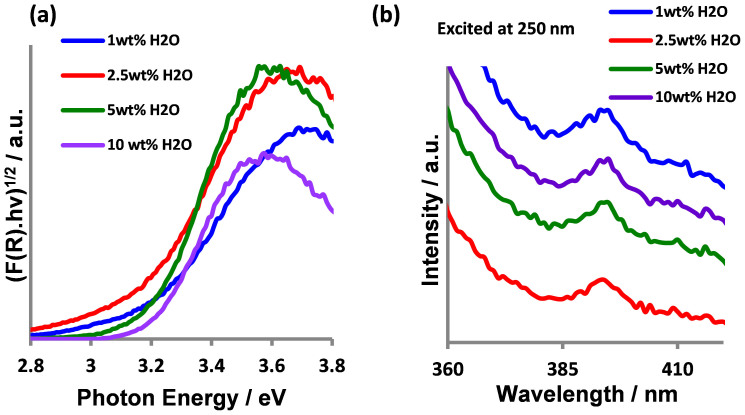
(a) UV-Vis absorption spectra based on Kubelka-Munk function vs. the photon energy and; (b) Photoluminescence spectra of the as-prepared α-MoO_3_ thin films over the wavelength range 360–420 nm.

Optical properties of the prepared α-MoO_3_ thin films were probed using UV-visible reflectance and photoluminescence (PL) spectroscopy. The optical band gap (E_g_) was estimated using (F(R).hν)^1/2^ vs. hν plots as shown in Figure 4a. The optical band gap is found to be ~3.2 eV for all samples, which is in good agreement with values reported in the literature^18^,^19^. The electronic structure of MoO_3_ can be described by a conduction band composed of dominant Mo 4d orbitals and a O 2p valence band region. Figure 4b shows the PL spectrum of the samples, where an emission band at around 393 nm is observed. The emission band, with an equivalent energy value of around 3.15 eV, corresponds reasonably well to the optical band gap of α-MoO_3_.

### Photocharging and Discharging of MoO_3_

**Figure 5:**
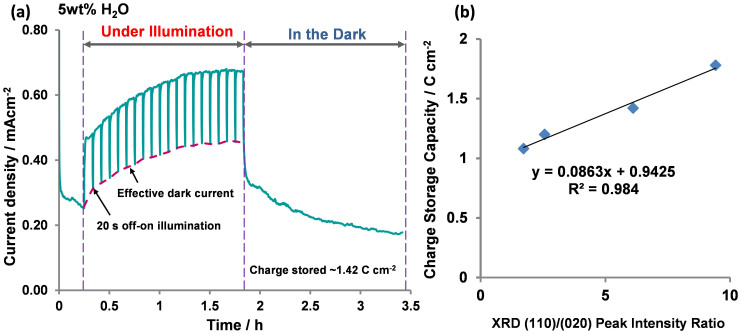
(a) Current profiles of α-MoO_3_ thin films (synthesised in 5 wt% H_2_O) during charging (illumination) and discharging (dark) processes. Applied potential, 1.0 V vs Pt; Electrolyte 0.1 M Na_2_SO_4_; UV-illumination source: 300 W Xe lamp. The red dotted line indicates development of the effective dark current during illumination. The area under the discharge curve indicates total amount of charge released in dark and, (b) linear relationship between change storage capacity and XRD I_110_/I_020_ peak intensity of α-MoO_3_ thin films.

Figure 5 shows the current profile of α-MoO_3_ (5 wt% water) under charging (illuminated) and discharging (dark) conditions. The effective dark current is defined as the current reached when illumination is temporarily switched off (for 20 s) during each off-on cycle. In this 20 s period, the effective dark current did not fall to the initial current level (i.e. the current recorded in the PEC cell prior to illumination) instead remaining at an elevated value. On repeated 20 s off-on cycles, a clear development in effective dark current is observed as represented by the red-dotted line in Figure 5. The presence of the effective dark current signifies a continual and undisrupted flow of excited charges from α-MoO_3_ to the external circuit despite the absence of illumination. Hence, the development of the effective dark current reflects the ability of α-MoO_3_ to accumulate and store a portion of the excited charges during illumination and discharge the stored electrons in the dark, akin to a self-photorechargeable PEC cell.

The MoO_3_-based PEC cell was continuously charged for 1.5 h under UV illumination and then discharged in the dark for the same duration. During the discharging process, the current gradually diminished with time, indicating the release of stored excited charges. The area under the discharge curve corresponds to the total amount of charge released in the dark with the charge storage capacities of α-MoO_3_ synthesised from the different electrolytes found to be: 1.08 C cm^−2^ (1 wt% H_2_O), 1.20 C cm^−2^ (2.5 wt% H_2_O), 1.42 C cm^−2^ (5 wt% H_2_O) and 1.78 C cm^−2^ (10 wt% H_2_O). On plotting the charge storage capacity against the XRD I_(110)_/I_(020)_ peak intensity ratio of the films (produced from the water content), a strong linear relationship is apparent as shown in Figure 5b. The strong linear relationship between charge storage capacity and XRD I_(110)_/I_(020)_ peak intensity ratio of the MoO_3_ films provides evidence that alkali cation intercalation is occurring at the (kk0) lattice planes.

### Proposed Charge/Discharge Mechanisms

The self-photo-recharge phenomenon of α-MoO_3_ has been found to occur under what are generally perceived as non-favourable conditions: (i) a positive bias, (ii) absence of a hole scavenger and (iii) no external source of electrons. Under a positive bias, electrons are continuously extracted from the MoO_3_ electrode. This makes the intercalation of positively-charged alkali cations into the α-MoO_3_ structure more challenging. In the absence of a hole scavenger, there is a higher probability for electron-hole pairs to recombine than accumulate. With no external source of electrons, any electron to be stored has to be generated by α-MoO_3_.

Previous Raman spectroscopy studies confirmed the light-induced charge storage and discharge mechanisms of α-MoO_3_ are related to the (de)intercalation of alkali cations into the α-MoO_3_ structure^14^. The self-photo-recharge mechanisms of α-MoO_3_ are represented by Eq. 3–7. Electron-hole pairs are formed in α-MoO_3_ upon photo-excitation (Eq. 3). Holes will oxidise water molecules that are absorbed on the film surface to produce oxygen and protons (Eq. 4). The negative electric field induced by the delocalised electrons facilitates the intercalation of positive cations (A^+^: H^+^ and Na^+^) from the electrolyte into the α-MoO_3_ structure, leading to the formation of molybdenum bronze, A_x_MoO_3_ (Eq. 5). While a portion of the photoexcited electrons are transported by the positive bias to the Pt electrode (where they participate in water reduction (Eq. 6), some of the photoexcited electrons are neutralised and stabilised within the α-MoO_3_ structure via the intercalated positive cations (Eq. 5). In the absence of illumination, the positive bias facilitates the discharge of stored charges from α-MoO_3_ with the discharge of electrons accompanied by a simultaneous de-intercalation of the positive cations (Eq. 7).


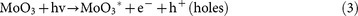

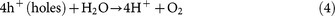

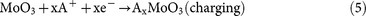

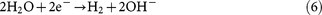

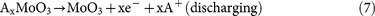


### Influence of Iso-oriented (0k0) and (kk0) Crystallography on the Self-Photo-Recharge Ability of α-MoO_3_

**Figure 6:**
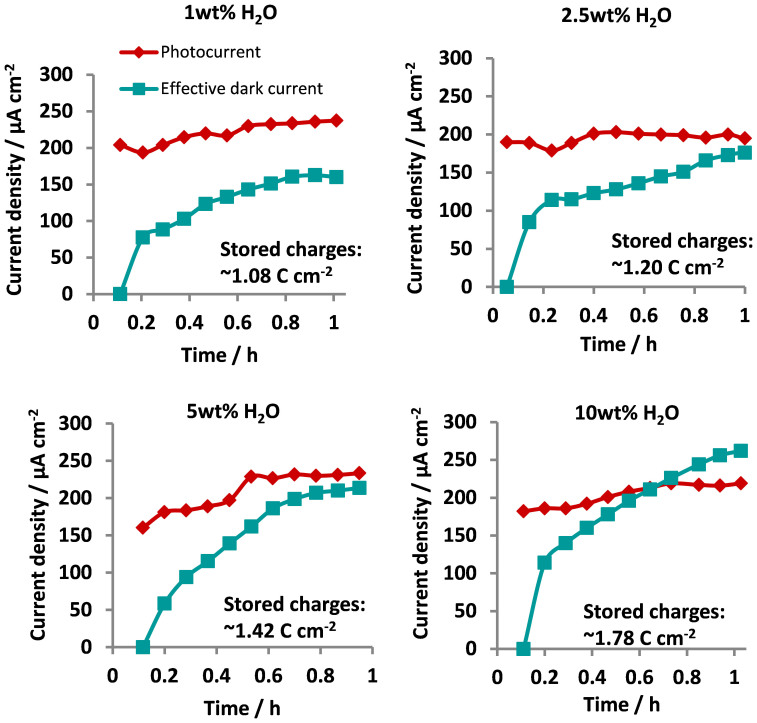
Effective dark current and photocurrent density profiles for α-MoO_3_ thin films synthesised using anodisation electrolytes with different water concentrations. Applied potential: 1.0 V vs Pt; Electrolyte: 0.1 M Na_2_SO_4_; UV-illumination source: 300 W Xe lamp.

Figure 6 shows the photocurrent and effective dark current profiles for α-MoO_3_ synthesised with varying water concentrations. The magnitude of the anodic photocurrent defines the rate of excited charge transfer from the (α-MoO_3_) anode to the (Pt) cathode under the positive bias while the magnitude of the effective dark current density indicates the total amount of excited charge stored with illumination time. As shown in Figure 6, the rates of anodic charge transfer are fairly consistent over the illumination period for each α-MoO_3_ thin film at ~200 μA cm^−2^. On the other hand, the total amount of charge stored is strongly time dependant, as implied from the gradual development of the effective dark current levels. Stabilisation of the effective dark current densities after approximately one hour of photo-charging indicates the α-MoO_3_ electrodes have attained their maximum charge storage capacities. Comparing the charge storage capacities across the α-MoO_3_ thin film samples further demonstrates a clear dependence of charge storage on the film crystallography. A much higher level of charge storage is achieved for films synthesised at higher water concentration due to the increasing presence of (kk0) planes for alkali cation intercalation.

If the relative portion of charge stored and transferred are compared between α-MoO_3_ crystals with dominant (kk0) planes in this work and the α-MoO_3_ crystals dominant in the (0k0) planes from previous work^14^, α-MoO_3_ films with a higher ratio of (kk0) planes exhibit a faster charging time. That is, α-MoO_3_ films with dominant (kk0) planes require approximately 50% less charging time compared to the (0k0)-oriented MoO_3_ crystals. The fast charging time of (kk0)-oriented MoO_3_ crystals is attributed to the narrower lattice gaps of the (kk0) planes [e.g. 3.81 Å for (110), JCPDS card no. 05-0508], which impedes the rate of alkali cation de-intercalation and promotes rapid stabilisation of the alkali cations within the layered framework. The lattice spacing of the (0k0) planes [e.g. 6.93 Å for (020), JCPDS card no. 05-0508] in contrast is significantly large owing to the weak interlayer interactions (i.e. the van der Waal's gap) along the [010] direction. Consequently, the small alkali cations can rapidly diffuse from the large van der Waal's gaps which in turn necessitates a longer duration for charges to build up in the (0k0) planes.

Comparing the level of charge storage shows α-MoO_3_ films with dominant (0k0) planes^14^ exhibit a better charge storage capacity than the (kk0)-oriented MoO_3_ crystals. The surface area-normalised charge storage capacity for the (0k0)-oriented MoO_3_ film^14^ was found to be 2.09 C cm^−2^ while the highest charge storage capacity achieved with the (kk0)-oriented MoO_3_ film (10 wt% H_2_O) was determined to be 1.78 C cm^−2^. Compared to the (kk0)-oriented MoO_3_ crystals, the layered crystalline structure of α-MoO_3_ crystals with dominant (0k0) planes is constructed from much larger octahedral sheets caused by the structural anisotropy in the [001] direction. The van der Waal's gaps of the (0k0)-oriented crystals therefore contain more active sites for the intercalation of alkali cations, leading to a better charge storage capacity.

Another significant feature observed in Figure 6 is the almost equal magnitudes of the anodic photocurrent and effective dark current densities for the different (kk0)-oriented MoO_3_ films at steady-state. In general, the (kk0)-oriented MoO_3_ films show the ability to store and release nearly equal proportions of the excited charges generated in the MoO_3_ photoanode during illumination. However, in the case of the (0k0)-oriented MoO_3_ thin film^14^ its steady-state effective dark current level strongly exceeded its photocurrent level. These results illustrate the selectivity of the α-MoO_3_ crystal planes for excited charge storage and release. α-MoO_3_ crystals with dominant (0k0) planes are highly effective for excited charge storage while the (kk0)-dominant MoO_3_ crystals can attain a strong balance between charge release for immediate usage under the illuminated condition and charge storage for subsequent utilisation in dark.

The ability of the (kk0)-oriented MoO_3_ films to release a significant portion of the excited charges as anodic photocurrent can also be attributed to the well-developed {hk0} and {0k0} crystal facets of the (kk0)-oriented MoO_3_ crystals as shown in Figure 2b. Recent studies on crystal facet engineering of semiconductor photocatalysts (e.g. TiO_2_, Fe_2_O_3_ and BiVO_4_) have demonstrated the selectivity of different crystal facets for photogenerated electrons or holes^20^,^21^,^22^. The unique atomic arrangements in different crystal facets have been proven to prompt subtle variations in the surface energy levels of the conduction and valence bands^21^,^23^. The small difference in energy levels is sufficient to drive electrons and holes to specific crystal faces, leading to better separation of electron-hole pairs which in turn contribute to photocatalytic enhancement.

## Conclusions

The results presented here illustrate the selectivity of α-MoO_3_ crystal planes for excited charge storage and release under illuminated and non-illuminated conditions. α-MoO_3_ crystals with dominant (0k0) planes showed a preference for excited charge storage. On the other hand, α-MoO_3_ crystals with iso-oriented (kk0) crystalline planes can achieve fast charging and a strong balance between charge release for immediate exploitation under illuminated condition and charge storage for subsequent utilisation in the dark. The ability to tune the crystallographic orientation of α-MoO_3_ crystalline layers by simple adjustment of the anodisation electrolyte composition to facilitate regulation between excited charge storage and transfer in α-MoO_3_ is clearly demonstrated.

## Author Contributions

S.N.L. and N.Y. fabricated the electrodes and conducted the experiments. R.A., J.S. and Y.H.N. supervised this work. All authors analysed the data and completed the paper.

## Supplementary Material

Supplementary Information Supporting Information - Influence of MoO_3_ (110) Crystalline Plane on Its Self-Charging Photoelectrochemical Properties

## Figures and Tables

**Figure 1:**
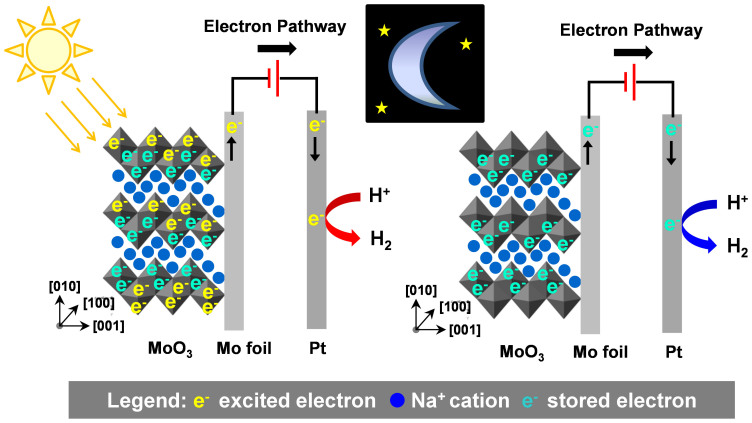
Schematic representation of self-photo-recharge ability of MoO_3_. Subjected to positive bias and illumination, MoO_3_ demonstrates the capacity to generate and store excited electrons in its layered structure through the intercalation of alkali cations. The stored electrons can be subsequently released under discharging conditions in the dark to allow reductive reaction(s) to continue to occur in absence of light.
