# Supplementary material for: Influence of MoO3(110) Crystalline Plane on Its Self-Charging Photoelectrochemical Properties
Source: Sci Rep. 2014 Dec 11;4:7428. doi: 10.1038/srep07428 (PMC4262818; doi:10.1038/srep07428)
Supplement: Supplementary Information — Supporting Information - Influence of MoO3 (110) Crystalline Plane on Its Self-Charging Photoelectrochemical Properties [file srep07428-s1.doc]

**Supporting Information**

Influence of MoO_3_(110) Crystalline Plane on Its Self-Charging Photoelectrochemical Properties

Shi Nee Lou, Nicholas Yap, Jason Scott, Rose Amal, Yun Hau Ng^*^

Particles and Catalysis Research Group, School of Chemical Engineering, The University of New South Wales, Sydney NSW 2052, Australia.

Figure S1 shows the SEM image of α-MoO_3_ thin film anodised in ethylene glycol electrolyte solution containing 10wt% H_2_O and 0.5wt% NaF. For clarification, the (kk0)-oriented crystalline layers of α-MoO_3_ is refer to the promoted growth along (kk0) planes within the crystal under current synthetic conditions. It did not imply the morphological anisotropic growth such as nanorods and nanotubes. Highly aligned nanostructure was, therefore, not within experimental expectation. Nonetheless, the presence of (kk0)-planes can be conveniently observed in almost every individual particle on the thin film as shown in Figure S1, where the magnified areas of the SEM images indicate the {kk0} facet.

Figure S1: SEM images of α-MoO_3_ thin film (10wt% water) and selected magnified areas indicating the {kk0} crystal facet. The scale bars on the magnified SEM images represent 250 nm.
